# Idiopathic peritonitis in horses: a retrospective study of 130 cases in Sweden (2002–2017)

**DOI:** 10.1186/s13028-019-0456-2

**Published:** 2019-04-25

**Authors:** Emma Odelros, Anna Kendall, Ylva Hedberg-Alm, John Pringle

**Affiliations:** 1 Mälaren Equine Clinic, Hälgesta 1, 193 91 Sigtuna, Sweden; 2 University Equine Hospital, Swedish University of Agricultural Sciences, Box 7040, 750 07 Uppsala, Sweden; 3 Department of Clinical Sciences, Swedish University of Agricultural Sciences, Box 7040, 750 07 Uppsala, Sweden

**Keywords:** Abdomen, Colic, Fever, Inflammation

## Abstract

**Background:**

Peritonitis in horses is historically associated with prolonged treatment regimens of broad-spectrum antimicrobials and a guarded prognosis for survival. The condition is most often seen as a secondary complication to traumatic injuries involving the abdominal cavity, rupture of bowel or abdominal surgery. However, cases of idiopathic peritonitis with no such underlying cause have been described. In Sweden idiopathic peritonitis is commonly identified and, in contrast to peritonitis secondary to traumatic incidents, affected horses appear to respond well to medical treatment. The objectives of this study were to describe clinical signs, laboratory findings, bacterial culture results, treatment regimens and survival rates for horses diagnosed with idiopathic peritonitis.

**Results:**

Medical records were obtained from horses diagnosed with peritonitis without identifiable cause. Diagnosis was based on macroscopically abnormal peritoneal fluid, with an elevated nucleated cell count (> 10 × 10^9^ cells/L) or total protein (> 25 g/L). A total of 130 horses were included, presenting with pyrexia (83%), lethargy (80%), anorexia (68%) and abdominal pain (51%). Microbial cultures were performed in 84% of the cases of which 41% were positive. The most commonly recovered bacteria were *Actinobacillus* spp., cultured from 21% of the submitted samples. All horses received antimicrobial therapy and many responded to treatment with penicillin alone. Survival until discharge was 94%.

**Conclusions:**

Idiopathic peritonitis is a disease that should be considered in horses presented with fever, signs of colic and lethargy. Medical treatment of idiopathic peritonitis is often successful and in Sweden most cases appear to respond well to treatment with penicillin as the sole antimicrobial.

## Background

Peritonitis is a well described illness in horses, often occurring as a secondary complication to traumatic injuries involving the abdominal cavity, rupture of bowel or abdominal surgery. Key clinical signs include colic, pyrexia and, in more chronic cases, weight loss [1, 2]. Diagnosis is based on evaluation of peritoneal fluid, with an elevated nucleated cell count being indicative of peritoneal inflammation [1–4]. Secondary peritonitis caused by trauma, abdominal surgery or neoplasia has historically been associated with prolonged treatment regimens with broad-spectrum antimicrobials [1–5]. Cases of secondary origin are often coupled to a guarded prognosis, with survival varying between 40 and 86% [2–5]. Cases of peritonitis without identified cause are termed primary or idiopathic peritonitis, with described survival rates between 57 and 94% (Table 1). Idiopathic cases have been associated with *Actinobacillus equuli* infection, and better outcomes have been reported in these horses [6–8]. Hypothesized causes of idiopathic peritonitis include leakage of gastrointestinal microorganisms from the gastrointestinal tract by migration of parasites or foreign bodies, mucosal erosions in the large intestine caused by treatment with non-steroidal anti-inflammatory drugs (NSAIDs) [9, 10] and non-strangulating intestinal infarctions [11]. Previous studies have either not differentiated between idiopathic and secondary cases, only included small numbers of true idiopathic peritonitis [1–5, 12] or solely described idiopathic cases associated with *A. equuli* infection [6–8]. In contrast to previous work, the aim of the present study was to include only idiopathic peritonitis and to describe the clinical signs, bacterial culture results, treatment regimens and survival rates for the disease.

**Table 1:** Survival rates for horses with peritonitis

Author [references]	Number of cases	Survival to discharge
Dyson [3]	30^a^	21/30 (70%)
Mair et al. [1]	21^a^	12/21 (57%)
Hawkins et al. [4]	28^a^	16/28 (57%)
Gay and Lording [6]	5^b^	4/5 (80%)
Golland et al. [8]	15^b^	15/15 (100%)
Matthews et al. [7]	51^b^	51/51 (100%)
Southwood and Russell [12]	55^a^	43/55 (78%)
Henderson et al. [2]	50^a^	47/50 (94%)
Nógrádi et al. [5]	12^a^	93%
Odelros et al. current data	130	122/130 (94%)

^a^Including horses with peritonitis secondary to dystocia, castration complications, rectal tears, perforated intestinal tract, intestinal strangulation, flank puncture wounds, uterine tears, abdominal abscesses, cholangiohepatitis and chronic hepatitis. Not possible to identify the number of true idiopathic cases in the data presented

^b^*Actinobacillus equuli* only

## Methods

Data were retrieved through a retrospective review of medical records from horses diagnosed with peritonitis at two referral hospitals in Sweden during 2002–2017. Included diagnose codes were “peritonitis” and “purulent peritonitis”. Horses that had a history of trauma or neoplasia resulting in peritonitis were excluded. Traumatic causes included uterine rupture, external trauma, recent abdominal surgery, ruptured bowel and castration complications.

Horses were included if the total cell count was increased above normal or, in cases where laboratory analysis was not performed, if the peritoneal fluid was visibly abnormal with an obvious change in color and turbidity. Cut-off values were selected for nucleated cell count in peritoneal fluid at > 10 × 10^9^/L and for total protein level at > 25 g/L [13]. Normal peritoneal fluid was defined as clear and pale yellow [13].

Data collected included age, breed, sex, duration of clinical signs and clinical findings at admission. Vital parameters such as mental status, heart and respiratory rates, mucous membrane appearance, rectal temperature, any abnormal rectal findings and presence of gastric reflux were recorded. Furthermore, gross appearance and analysis of peritoneal fluid including leukocyte count and total protein, cytological findings and bacterial culture results were retrieved when available. For most horses, a complete blood count (CBC) was performed at admission. These data were also included together with concentrations of serum amyloid-A (SAA), plasma fibrinogen, serum total protein and albumin at presentation. In some cases, a follow-up SAA was available. SAA was determined using LZ test SAA (Eiken Chemical Co, Tokyo, Japan). In a small number of cases seen at one of the hospitals, StableLab (Epona Biotech Ltd, Sligo, Ireland) was used. Follow-up samples were analyzed using the same method as for previous samples from the same horse. Fibrinogen was determined using K-assay Fibrinogen (Kamiya Biomedical Copmpany, Seattle, WA, USA) or the QBC-Vet Autoreader (IDEXX, Westbrook, ME, USA).

When presented in the records, fecal egg counts and history of anthelminthic treatment were included. The peritonitis treatment regimen, length of hospitalization and outcome was included for each case. Long-term follow-up, for more than 12 months after discharge, was obtained by telephone interviews with owners.

## Results

A total of 251 cases were found in the initial search. Three horses were euthanized at admission without further investigation and were therefore excluded. Four horses diagnosed with eosinophilic peritonitis were excluded, as well as 113 horses diagnosed with peritonitis secondary to trauma, foaling complications, surgery or neoplasia. One horse was excluded due to concurrent bacteremia and meningitis. Of the 130 horses that remained in the final study group diagnosed with idiopathic peritonitis, 70 (54%) were geldings, 48 (37%) mares and 12 (9%) stallions. Ages ranged from six months to 30 years (mean age 11 years, median 10 years). Breeds represented included 51 (39%) Warmbloods, 25 (19%) Icelandic horses, 10 (8%) Standardbreds, 5 (4%) Thoroughbreds, 17 (13%) were of various other breeds and 22 (17%) of various pony breeds. Demographics were reflective of the hospital populations during the same time period.

Admission dates were distributed throughout the year, 38 horses (29%) were presented in the winter between December and February, 33 (26%) in spring between March and May, 21 (16%) in the summer between June and August and 38 (29%) in autumn between September and November.

Clinical data and laboratory values from the included horses are presented in Table 2. Most horses were examined by a veterinarian in the field prior to hospital admission, and 55 (42%) had received treatment with metamizole and/or flunixin meglumine prior to referral.

**Table 2:** Clinical findings and laboratory variables from horses with idiopathic peritonitis

Clinical variable	Number of horses (%)	Mean value (range)
Duration of clinical signs prior to admission (days)		3 (1–56), median 1 day
Lethargy	104 (80)	
Colic	92 (51)	
Pyrexia (°C)	108 (83)	38.6 (37–40.8)
Heart rate/min		52 (30–120)
Resp rate/min		21 (8–72)
Endotoxemia [13]	38 (29)	

	Horses sampled (%)	Mean value (range)
Peritoneal fluid		
Lactate (mmol/L)	46 (35)	7 (0.8–19)
Nucleated cell count (/L)	123 (95)	172 × 10^9^ (13–620 × 10^9^)
Neutrophil proportion (%)	123 (95)	89 (72–99)
Total protein (g/L)	97 (75)	43 (18–72)
Cytological examination	88 (68)	
Bacteria seen on cytology	15/88 (17)	
Laboratory variables		
SAA (mg/L)	98 (75)	799 (90–2754)
Fibrinogen (g/L)	98 (75)	5 (2–11)
Serum total protein (g/L)	55 (42)	66 (36–77)
Serum albumin (g/L)	50 (38)	34 (20–38)
CBC performed	119 (92)	
Normal leukogram	50/119 (42)	
Normal cell count with left shift (> 1% bands)	22/119 (19)	
Leukocytosis (> 12.5 × 10^9^/L)	17/119 (14)	
Leukopenia (< 5.5 × 10^9^/L)	30/119 (25)	
Fecal egg count	49 (38)	
Positive egg count	43/49 (88)	
Unspecified strongyles (EPG)	18	608 (50–1050)
Cyathostomes (EPG)	12	283 (50–1050)
Unspecified strongyles (EPG) and *Anoplocephala perfoliata*	4	838 (50–1050)
*Ascaris equorum* (EPG)	3	633 (50–1050)
*Anoplocephala perfoliata*	2	No EPG values available

A per-rectal examination was performed at admission or during the following day in 126 (97%) of the horses. Abnormal rectal findings consistent with an impaction in the large colon were found in 50/126 (40%) and was the only abnormality found on per-rectal palpation.

Abdominocentesis was performed in all cases, of which 129 (99%) horses had macroscopically abnormal peritoneal fluid with a change in color and/or increased turbidity. Noted abnormalities in color were dark yellow, orange, red–orange and red. For one horse, no visual assessment of the peritoneal fluid was noted in the medical record, but the fluid had a markedly increased nucleated cell count.

Microbial culture was performed in 109 (84%) of the cases. The majority of samples were processed for both aerobic and anaerobic culture, with 67/109 (61%) bottled in blood culture medium and 42/109 (39%) in sterile plastic collection tubes. Bacteria were cultured from 45/109 (41%) of the submitted samples. Of the samples submitted in blood culture medium, 36/67 (54%) were positive. Of the samples submitted in sterile plastic collection tubes, bacteria were cultured from 9/42 (21%). The most commonly found bacteria were *Actinobacillus* spp., cultured from 23/109 (21%) of the samples. Strains of *A. equuli, A. suis* and *Actinobacillus suis*-like were represented, but 14/23 of the *Actinobacillus* cultures were not further subtyped. Betahemolytic streptococci were found in 8/109 (7%) of the cultured samples, *Bacteroides* spp. in 5/109 (5%), *Escherichia coli* in 3/109 (3%), *Fusobacterium necrophorum* in 5/109 (5%), *Streptococcus gallolyticus* in 1/109 (1%) and alphahemolytic streptococci in 1/109 (1%). Of the positive cultures, 39/45 (86%) were pure culture and 3/45 (7%) included more than one type of bacteria. The samples with mixed growth were all submitted in blood culture medium and included strains of *Actinobacillus* spp., *Fusobacterium* spp. and *Bacteroides* spp. For the remaining 3/45 (7%) positive cultures, unspecified growth was noted. A total of 64/109 (59%) submitted cultures were negative.

In the antimicrobial susceptibility tests, all strains of *Actinobacillus*, *Streptococcus* and *Fusobacterium* cultured were susceptible to penicillin. The *E. coli* cultured was susceptible to gentamicin in all three horses, but only to trimethoprim-sulphadiazine in two of them. *Bacteroides* spp. was not subtyped in any of the positive cultures and no antimicrobial susceptibility test was performed. Based on the performed antimicrobial susceptibility tests, at least 29/45 (64%) of the cultured bacteria were susceptible to penicillin.

A follow-up SAA was analyzed to facilitate the decision of when to discontinue treatment in 94 (72%) of the horses, with the final result varying from 0 to 900 mg/L (mean 289 mg/L, median 345 mg/L).

Flunixin meglumine was administered to 129 (99%) of the horses as an anti-pyretic drug, for pain relief and with the aim to treat or prevent endotoxemia. A total of 122 (94%) horses received intravenous fluid therapy with acetated ringers, three of these horses had glucose supplementation to their fluids and one horse received additional 20 mEq intravenous potassium chloride per liter of intravenous fluids.

**Fig. 1:**
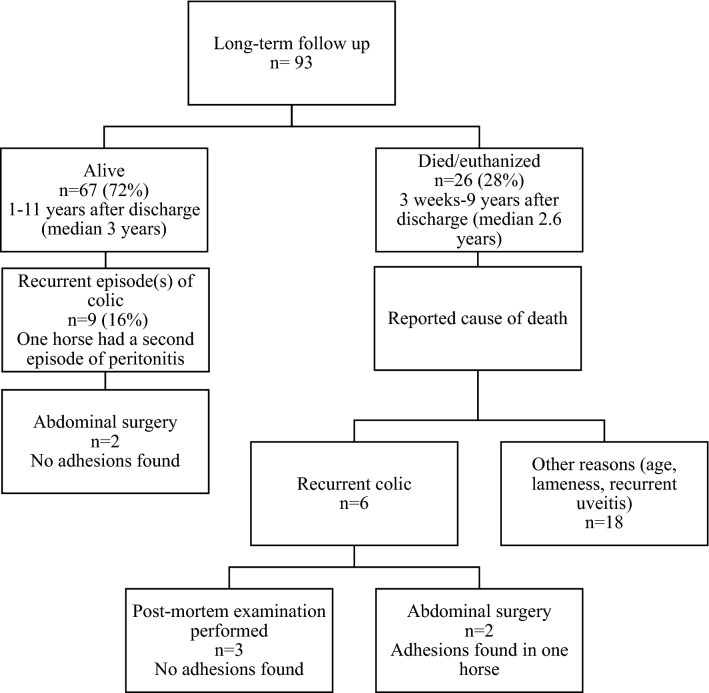
Long-term follow up of discharged horses

All horses received antimicrobial therapy. In the middle of the study period, a new treatment protocol for idiopathic peritonitis was implemented at the contributing hospitals. Based on observations, clinical experience and antimicrobial resistance patterns in frequently cultured bacteria in peritonitis cases, a decision was made to initiate monotherapy with penicillin. If the horse was presented with signs of endotoxemia [14] or did not respond to treatment within 24 h, the treatment regimen was extended to also include gentamicin or trimethoprim-sulphadiazine at the discretion of the attending clinician. Treatment with penicillin as the only antimicrobial was initiated in 47 (36%) of the horses, 43 (91%) of which responded very well to this treatment. In four of the penicillin-treated horses, the regimen had to be extended due to lack of response to treatment and antimicrobial susceptibility results. In summary, 43 (33%) were treated with penicillin solely, 76 (59%) were treated with a combination of penicillin and gentamicin, 5 (4%) with penicillin and trimethoprim-sulphadiazine, 4 (3%) with penicillin, gentamicin and trimethoprim-sulphadiazine and 2 (1%) were treated with a combination of penicillin, gentamicin and metronidazole. Dosages used were sodium penicillin G 10,000–20,000 IU/kg IV q6h to q8h, procaine penicillin G 20,000 IU/kg IM q24h, gentamicin 6.6 mg/kg IV q24h, trimethoprim-sulphadiazine 30 mg/kg PO q12h or 24 mg/kg IV q12h and metronidazole 15 mg/kg PO q8h. The total number of treatment days were known for 119/122 (98%) survivors and varied between 5 and 30 days (mean 13 days and median 11 days). The hospitalization time for survivors varied between 4 and 25 days (mean and median 10 days). A total of 122 (94%) horses survived until discharge. Eight (6%) horses were euthanized, six of these due to lack of response to treatment in combination with financial constraints after 2, 2, 4, 4, 10, and 12 days respectively. A post-mortem examination was performed in one horse, revealing a diffuse peritonitis with multiple abscesses in the mesentery and large numbers of adult *Parascaris equorum* in the small intestine. One horse developed laminitis during the hospital stay and was euthanized on day 18. A second horse developed signs of colic due to presumed abdominal adhesions when treatment was discontinued. The horse was euthanized due to poor prognosis but unfortunately no post-mortem examination was performed. Long-term follow-up was available for 93/122 horses (76%), of which 90/93 (97%) survived > 12 months after discharge. Long-term follow-up details are presented in Fig. 1.

## Discussion

This study is the first to present a large number of cases with idiopathic peritonitis associated with various bacteria. Medical treatment was found to be successful in most idiopathic cases, independent of the bacteria cultured. Moreover, many horses responded to antimicrobial treatment with penicillin alone.

Horses with peritonitis are often presented with lethargy, fever and abdominal pain. The onset of clinical signs in idiopathic cases is often acute, without previous history of illness. Although it was a common clinical finding in this study, the incidence of pyrexia is likely to be underestimated since almost half of the included horses received NSAIDs prior to referral. The mean duration of clinical signs before hospital admission was 3 days, but one of the horses included was presented with a history of 56 days, during which it had four recurrent episodes of colic and fever. Excluding this horse, the mean duration of clinical signs before hospital admission was 1 day.

Since the CBC and plasma fibrinogen can be normal in horses with idiopathic peritonitis, these are not reliable parameters for confirming or ruling out the diagnosis. SAA is often markedly elevated in peripheral blood in affected cases. This, in combination with the clinical presentation described above, should alert the clinician to the need for performing an abdominocentesis. In cases of idiopathic peritonitis, the peritoneal fluid is turbid with or without a change in color, with an elevated nucleated cell count and total protein concentration. Previously described cut-off values for nucleated cells in peritoneal fluid range between 5 and 10 × 10^9^/L [1–3, 7] but normal horses have been found to have up to 10 × 10^9^ nucleated cells/L in peritoneal fluid [13]. In the present study, a cut-off value of 10 × 10^9^/L was chosen as a defensible definition of peritoneal inflammation and to increase the specificity of included cases.

Consistent with findings in previous studies, bacteria could not be cultured from all peritoneal fluid samples from horses with peritonitis despite suspected bacterial etiology [1, 3, 7]. Cultures in this study were positive in 41% of the submitted samples, but almost half of the samples were submitted for anaerobic culture in sterile plastic tubes which could have impaired the bacterial growth, either due to the lack of culture medium or to an excess of oxygen. A similar situation is described in human medicine, where positive cultures have been reported in 58–75% of patients with septic peritonitis, with higher sensitivity for samples in blood culture medium [15]. Poor bacterial growth has also been noted in horses with suspected synovial sepsis, where the proportion of positive cultures has been reported to be 72% despite clinical findings consistent with a septic process [16]. Reasons for not performing a bacterial culture in horses in the present study were not noted in the records but could be that the amount of fluid obtained was insufficient or coagulated. Bottles containing blood culturing media are more expensive and this could be an argument for using sterile plastic tubes instead. However, the results from this study highly suggest that blood culture media should be used if possible, to increase the sensitivity of the culture.

The microbiological methodology is a limitation of this retrospective study, as culture and subtyping techniques have been developed further during the study period, and new bacterial subtypes have been identified over time. This complicates comparisons of culture results within the study period, but also with results from other studies.

Of the positive cultures obtained in this study, a majority were *Actinobacillus* spp. *A. equuli* was cultured from several horses, but strains of *A. suis* and *A. suis*-like were also found. The clinical relevance of these species remains unknown but is not likely to be significant since all horses with *Actinobacillus* spp. peritonitis responded well to penicillin treatment. *Bacteroides* spp. were not subtyped in any of the positive cultures, the bacteria is historically thought to be susceptible to penicillin in horses in Sweden [17], and no antimicrobial susceptibility test was performed for this reason. Recently, penicillin resistant strains of *Bacteroides* spp. have been found in cultures from horses in Sweden, indicating that *Bacteroides* spp. should be subtyped and the antimicrobial susceptibility determined for every positive culture of the bacteria [17]. Important to note is that antimicrobial resistance in veterinary medicine is described as low in Sweden [18], and that the response to treatment may be reflected by this national setting and cannot be assumed to be true for all countries.

Traditionally, cases of idiopathic peritonitis in Sweden have been treated with a combination of intravenous penicillin and gentamicin until the plasma fibrinogen levels returned to normal. In the middle of the study period, a treatment protocol based on penicillin monotherapy as a first line choice was initiated. In addition, follow-up evaluation of SAA rather than fibrinogen was implemented to determine when treatment could be discontinued, based on the early response and the short half-life of SAA [19, 20]. In conclusion, this resulted in reduced use of broad-spectrum antimicrobials and shorter hospital stays, since horses that responded to penicillin could be discharged earlier and continue on intramuscular procaine penicillin treatment at home.

Based on the above findings, horses diagnosed with idiopathic peritonitis in Sweden or countries with similar antimicrobial resistance patterns can initially be treated with penicillin as the only antimicrobial. However, it is important to note is that increased minimum inhibitory concentration (MIC) values for penicillin have been observed in equine strains of *Actinobacillus* spp. [21]. For this reason, treatment should be initiated with intravenous penicillin q6h until culture results have been obtained, since *Actinobacillus* spp. is most often cultured from these horses. In cases that do not respond to treatment within 24 h, or if severe signs of endotoxemia or sepsis are present, broad-spectrum antimicrobial treatment should be considered.

The follow-up SAA was used differently among clinicians. The most common approach was to take a follow-up SAA at home after hospital discharge, and when the concentration had returned to normal, a confirming peritoneal sample was obtained to make sure that the treatment was not discontinued until the nucleated cell count and cytology of the peritoneal fluid were considered normal. However, some clinicians were satisfied with a notable decrease in SAA before hospital discharge and some horses were therefore discharged with intramuscular penicillin injections for a period of time before the treatment was discontinued without further blood- or peritoneal fluid samples performed. This explains the diversity in the follow-up SAA concentrations, as it was left to the clinicians’ discretion to discontinue treatments or discharge patients without further testing. The use of two different methods for determining SAA is a limitation of this retrospective study, as comparison of results between them is not recommended [22]. For this reason, treatment recommendations based on specific concentrations of SAA cannot be established from the given data. Since horses were discharged before treatment was discontinued, data on the total number of treatment days were not available for all horses.

The fact that a post-mortem examination was only performed in one of the non-survivors is a major weakness of this study, as we cannot confirm that the remaining non-survivals were truly idiopathic cases. It is possible that these horses had a secondary peritonitis, with underlying causes never identified. The non-survivals were all considered idiopathic cases at the time of euthanasia, and for this reason they were included in the study population even if post-mortem examinations were not performed. If these cases were in fact horses with secondary peritonitis, the prognosis for survival after idiopathic peritonitis is underestimated in this paper.

Seventy-two percent of the discharged horses were still alive as the long-term follow-up was conducted and 97% survived > 12 months. Long-term survival rates of 58–84% have previously been reported [2, 12]. The recurrence rate of idiopathic peritonitis seems to be very low, as a second episode was only reported for one horse in this study. After discharge, 17% of the horses suffered from recurrent episodes of colic, compared to a reported recurrence rate of 28% in horses treated medically for various other types of colic [23]. Based on this, horses treated for idiopathic peritonitis do not seem to be at increased risk of suffering from future colic episodes, compared to horses previously treated medically for various other types of colic.

The cause of the bacterial translocation in idiopathic peritonitis is still unknown, but considering the bacteria found they are likely to originate from the alimentary tract [24, 25]. In a retrospective study of horses diagnosed with peritonitis of unknown origin, necrosis or perforation of the gastrointestinal tract was found in all but one of the non-survivals [1]. The cause of the disease is difficult to determine in survivors, but the mucosal barrier in the intestine is likely to be temporarily compromised to some extent, allowing temporary translocation of bacteria. A suggested cause of bacterial translocation is parasite migration [6–10] and interestingly, *A. equuli* has been isolated from verminous aneurysms of cranial mesenteric arteries caused by *Strongylus vulgaris* [26]. Peritonitis has also been diagnosed in horses subsequently found to have underlying non-strangulating intestinal infarctions caused by *S. vulgaris* [11]. Horses with infarctions do not respond to medical treatment and based on this finding, exploratory laparotomy has been proposed as an important intervention when peritonitis of unknown cause is diagnosed [11]. As horses with idiopathic peritonitis in Sweden respond well to medical treatment, they are not likely to suffer from extensive non-strangulating infarctions, but healing of small areas of infarction caused by migrating *S. vulgaris* have been suggested [1, 11] and is a possible underlying cause. The prevalence of *S. vulgaris* has been described as dramatically reduced after decades of frequent deworming, but implementation of selective anthelmintic therapy over the last years may be associated with a reoccurring increase in the prevalence of the parasite [27]. The seasonal distribution in the present study, with a lower incidence during the summer months, is similar to the seasonal pattern seen in horses with non-strangulating infarctions [11]. If this is incidental or related to lower burdens of hypobiotic and migrating parasites during summer months remains speculative.

Due to the previously suggested correlation between *S. vulgaris* and peritonitis, a positive fecal egg count in 88% of the sampled horses in this paper is an interesting finding. However, the relevance of this remains unclear since no horse had *S. vulgaris* infection confirmed and this further emphasizes the importance of performing a fecal polymerase chain reaction analysis or larval culture to identify *S. vulgaris* when strongyle eggs are found. Larval cyathostominosis has also been discussed as a potential cause of peritonitis, but has not been investigated further [2, 28]. It is important to remember that a negative fecal egg count does not rule out parasite infection, since migrating and encysted larvae are not producing eggs. Serology can facilitate diagnosis of prepatent infection by detecting *S. vulgaris*-specific antibodies [29] and a more widespread use of this diagnostic method could provide important insights when investigating the relationship between migrating parasites and idiopathic peritonitis further. Anthelmintic treatment has been recommended in horses diagnosed with peritonitis [6–8] and until proven otherwise, this is still a valid recommendation.

Large colon impactions were diagnosed at admission in almost half of the examined horses, often referred to as secondary impactions due to dehydration and pyrexia. However, the opposite cannot be ruled out, with intestinal leakage of bacteria due to compromised mucosa resulting from an impaction.

## Conclusions

Idiopathic peritonitis is a disease that should be considered in horses presented with fever, signs of colic and lethargy. Macroscopic and/or cytological changes of peritoneal fluid can confirm the diagnosis and a bacterial culture is a helpful adjunct if the horse is not responding to initial treatment. Medical treatment of idiopathic cases in this study was successful and, in Sweden, most infections respond to treatment with penicillin as the only antimicrobial.

## Abbreviations

CBC: complete blood count
NSAID: non-steroidal anti-inflammatory drug
SAA: serum amyloid A
